# Practice and Predictors of Do-Not-Resuscitate Orders in a Tertiary-Care Intensive Care Unit in Saudi Arabia

**DOI:** 10.1155/2024/5516516

**Published:** 2024-05-06

**Authors:** Abdulrahman Asiri, Farhan Zayed Alenezi, Hani Tamim, Musharaf Sadat, Felwa Bin Humaid, Wedyan AlWehaibi, Hasan M. Al-Dorzi, Yasir Adnan Alzoubi, Samiyah Alrawey Alanazi, Brintha Naidu, Yaseen M. Arabi

**Affiliations:** ^1^College of Medicine, King Saud bin Abdulaziz University for Health Sciences, King Abdullah International Medical Research Center, Intensive Care Department, King Abdulaziz Medical City, Ministry of National Guard-Health Affairs, Riyadh, Saudi Arabia; ^2^American University of Beirut Medical Center, Clinical Research Institute, Beirut, Lebanon; ^3^AlFaisal University, College of Medicine, Riyadh, Saudi Arabia; ^4^King Abdullah International Medical Research Center, Intensive Care Department, King Abdulaziz Medical City, Ministry of National Guard-Health Affairs, Riyadh, Saudi Arabia; ^5^King Saud bin Abdulaziz University for Health Sciences, King Abdullah International Medical Research Center, Intensive Care Department, King Abdulaziz Medical City, Ministry of National Guard-Health Affairs, Riyadh, Saudi Arabia

## Abstract

**Introduction:**

The objective of this study was to describe Do-Not-Resuscitate (DNR) practices in a tertiary-care intensive care unit (ICU) in Saudi Arabia, and determine the predictors and outcomes of patients who had DNR orders.

**Methods:**

This retrospective cohort study was based on a prospectively collected database for a medical-surgicalIntensive CareDepartment in a tertiary-care center in Riyadh, Saudi Arabia (1999–2017). We compared patients who had DNR orders during the ICU stay with those with “full code.” The primary outcome was hospital mortality. The secondary outcomes included ICU mortality, tracheostomy, duration of mechanical ventilation, and length of stay in the ICU and hospital.

**Results:**

Among 24790 patients admitted to the ICU over the 19-year study period, 3217 (13%) had DNR orders during the ICU stay. Compared to patients with “full code,” patients with DNR orders were older (median 67 years [Q1, Q3: 55, 76] versus 57 years [Q1, Q3: 33, 71], *p* < 0.0001), were more likely to be females (43% versus 38%, *p* < 0.0001), had worse premorbid functional status (WHO performance status scores 4-5: 606[18.9%] versus 1894[8.8%], *p* < 0.0001), higher prevalence of comorbid conditions, and higher APACHE II score (median 28 [Q1, Q3: 23, 34] versus 19 [Q1, Q3: 13, 25], *p* < 0.0001) and were more likely to be mechanically ventilated (83% versus 55%, *p* < 0.0001). Patients had DNR orders were more likely to die in the ICU (67.8% versus 8.5%, *p* < 0.0001) and hospital (82.4% versus 18.1%, *p* < 0.0001). On multivariable logistic regression analysis, the following were associated with an increased likelihood of DNR status: increasing age (odds ratio (OR) 1.01, 95% confidence interval (CI) 1.01–1.02), higher APACHE II score (OR 1.09, 95% CI 1.08–1.10), and worse WHO performance status score. Patients admitted in recent years (2012–2017 versus 2002–2005) were less likely to have DNR orders (OR 0.35, 95% CI 0.32–0.39, *p* < 0.0001). Patients with DNR orders had higher ICU mortality, more tracheostomies, longer duration of mechanical ventilation and length of ICU stay compared to patients with with “full code” but they had shorter length of hospital stay.

**Conclusion:**

In a tertiary-care hospital in Saudi Arabia, 13% of critically ill patients had DNR orders during ICU stay. This study identified several predictors of DNR orders, including the severity of illness and poor premorbid functional status.

## 1. Introduction

Determination of goals of care in seriously ill patients aims at providing goal-concordant care based on patient prognosis and values [1]. Despite being variably accepted, withholding and withdrawing life-sustaining measures frequently precede death in the ICU, [2–4] with the do-not-resuscitate (DNR) order being the commonest form of withholding life support. A systematic review of 56 studies on withholding or withdrawing life support in the ICU setting found that the mean prevalence of withdrawal of life-sustaining treatment for patients who died was 42.3% (standard deviation of 23.7%) and ranged from 0 to 84.1% [5]. The mean prevalence for withholding of life-sustaining treatment was 27.3% (standard deviation 18.5%) and ranged from 5.3 to 67.3% [5]. Many factors affect the determination of goals of care. These factors include the prognosis of the disease, severity of illness, patient preferences and physician opinions [5, 6]. In addition, institutional and societal norms and values may affect the determination of goals of care, leading to variability between institutions, countries and cultures [2–5, 7–9].

There is a growing enthusiasm to understand and improve decision-making regarding the goals of care in ICU patients. Most of the published studies about the determination of care goals in ICU patients come from Europe and North America [2–6]. There is a lack of studies from the Middle East, including Saudi Arabia [7, 9, 10]. Data from the international Intensive Care Outcome Network study (9524 patients) showed that 29 out of 354 patients (8.2%) from Middle Eastern countries, that included Saudi Arabia, had a decision to withhold/withdraw life-sustaining treatments [9]. This rate was significantly lower than that of Western Europe, even though the patients in Saudi Arabia had higher illness severity [9]. A one-year prospective study at our ICU (November 2009 and October 2010) found that 77% of 176 patients who died in the ICU had their goals of care determined, with 66% having DNR orders, 30% having withholding of life support and 4% having withdrawal of life support [11]. These decisions were made after a median time of four days after ICU admission [11]. In the present study, we evaluated the predictors and outcomes of patients with DNR orders during their stay in the ICU.

## 2. Materials and Methods

### 2.1. Study Design, Participants and Setting

This was a retrospective study of all patients admitted to the Intensive Care Department of King Abdulaziz Medical City, Ministry of National Guard-Health Affairs, Riyadh, Saudi Arabia, from 1999 to 2017. The hospital was a tertiary-care center with >1000 beds. The Intensive Care Department had multiple non-cardiac ICUs that functioned as closed units and admitted various medical, surgical and trauma patients. Board-certified intensivists covered the ICUs on a 24-hours per day, 7 days per week basis [12]. Multidisciplinary rounds were performed daily. Families were updated daily using an open visitation policy. Patients admitted to the ICU were “full code” on admission as the ICU admission criteria excluded patients who already had DNR orders. The goals of care were addressed at the discretion of the treating ICU team. Additionally, the patient's goals of care could be changed from “full code” to limited support, which included a DNR order, if three qualified physicians, including the most responsible/admitting attending physician, agreed that the patient would not benefit from continuation of “full code.” The goals of care were then discussed with the patient or surrogate decision-maker. If the patient or surrogate decision-maker disagreed, full support was usually provided. An established ethics committee could be consulted in case of conflict. Advance care directives, euthanasia and physician-assisted suicide were not practiced in our institution or in Saudi Arabia. This study was approved by the Institutional Review Board of the Ministry of National Guard -Health Affairs, Riyadh. Due to the study's retrospective nature, informed consent from patients/surrogate decision-makers was waived. We included the first ICU admissions for patients who were admitted more than once.

### 2.2. Data Collection

The study data were extracted from the departmental database which was maintained by a trained data collector since 1999. We recorded the demographic information, clinical data related to preadmission functional status based on WHO performance status score [13], admission diagnosis, sepsis at admission, ischemic and hemorrhagic stroke, chronic co-morbid conditions, Glasgow coma scale on admission, Acute Physiology and Chronic Health Evaluation (APACHE) II score, pertinent laboratory findings at ICU admission, and interventions during ICU admission, including mechanical ventilation and renal replacement therapy, and admission year.

The primary outcome of this study was hospital mortality. The secondary outcomes included ICU mortality, tracheostomy, duration of mechanical ventilation, and length of stay in the ICU and hospital.

### 2.3. Statistical Analysis

Patients were grouped into those who had DNR orders during ICU stay and those who were managed as “full code.” Frequencies and percentages were used to describe categorical variables, whereas medians and interquartile ranges (Q1, Q3) were used to present continuous variables. Pearson Chi-square was used to compare categorical variables between the two groups. Student's *t*-test was used to compare continuous variables. Binary logistic regression was performed to assess the independent factors associated with DNR orders. The following variables were entered into the model: age, pre-morbid WHO performance status, admission category, sepsis, ischemic stroke, hemorrhagic stroke, chronic cardiovascular disease, chronic renal disease, chronic respiratory disease, APACHE II score and admission year. Results were reported as odds ratio (OR) and 95% confidence interval (CI). The study sample size (24790 patients, 13% with DNR status) had at least 85% power to detect variables that increase or decrease the risk of DNR status by 20% with a type I error of 0.05 and a variable exposure rate in patients without DNR orders of at least 10%. Statistical software SAS version 9.0 (SAS Institute, Cary, NC, USA) was used for all analyses with two-tailed tests and an alpha error of 0.05. A *p* value <0.05 was considered to be statistically significant.

## 3. Results

### 3.1. Characteristics of Patients

Table 1 describes the characteristics of the study patients. During the study period, 24790 patients were admitted to the ICU, with 3217 (13%) patients having DNR orders during their ICU stay. The median time to the decision for DNR was three days (Q1, Q3: 1, 10). Table 1 shows the baseline characteristics of patients with a comparison of patients with DNR status versus patients with “full code.” Compared to patients with “full code,” ,patients with DNR orders were older (median 67 years [Q1, Q3: 55, 76] versus 57 years [Q1, Q3: 33, 71], *p* < 0.0001), and more likely to be females (1374 patients [43%] versus 8200 patients [38%], *p* < 0.0001), had a higher prevalence of comorbid conditions (except for chronic respiratory disease) and worse pre-morbid baseline functional status (WHO Performance Status scores 4-5: 606patients [18.9%] versus 1894patients [8.8%], *p* < 0.0001), had higher APACHE II score (median 28 [Q1, Q3: 23, 34] versus 19 [Q1, Q3: 13, 25], *p* < 0.0001) and were more likely to receive mechanical ventilation (2660 patients [83%] versus 11871 patients [55%], *p* < 0.0001). Sepsis was the most frequent reason for ICU admission (9488/24790 patients [38.3%]) and was more common in patients with DNR orders (47.8% versus 36.9%, *p* < 0.0001). Renal replacement therapy was also used significantly more in DNR patients (29.8% versus 12.7%, *p* < 0.0001).

**Figure 1:**
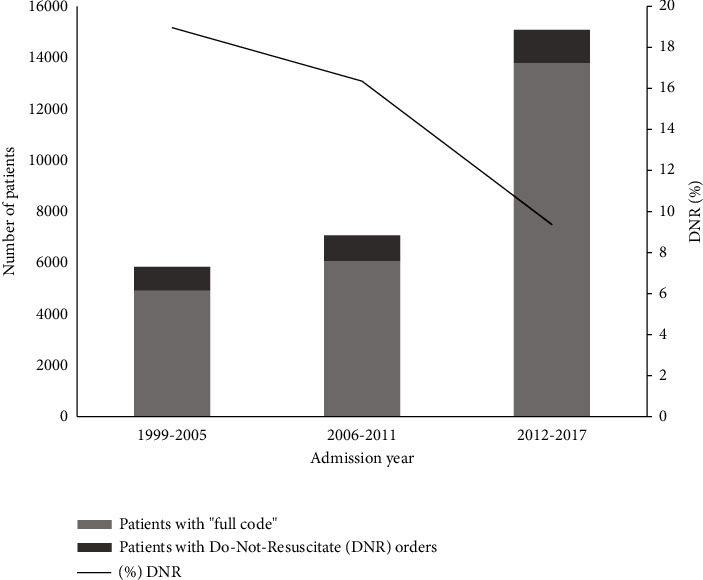
Number of patients who had and did not have do-not-resuscitate orders in different time periods (primary *Y* axis). The secondary *Y* axis shows the percentage of patients with do-not-resuscitate orders.

**Figure 2:**
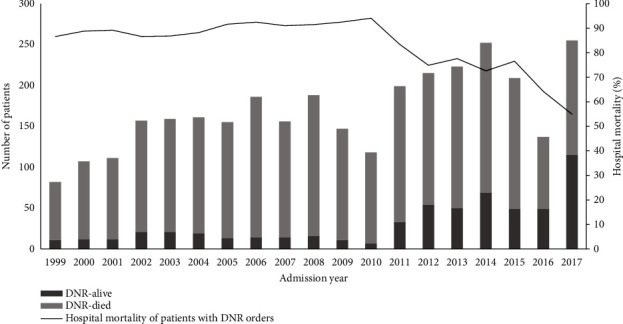
Number of survivors and nonsurvivors among the study patients who had do-not-resuscitate orders (primary *Y* axis). The secondary *Y* axis shows the mortality rate (percentage).

The number of admissions per year increased over the study period. Patients with DNR orders represented 19.81% of the patients admitted in the 1999–2005 period, 16.4% in the 2006–2011 period and 9.4% in the 2012–2017 period (Table 1 and Figures 1 and 2).

### 3.2. Predictors of DNR Order

Table 2 shows the predictors of DNR order (by multivariable logistic regression analysis). Older age (OR per year increment 1.01, 95% CI 1.009–1.015), worse pre-morbid WHO Performance Status at baseline, higher APACHE II score at admission (OR per unit increment 1.09, 95% CI 1.08–1.10) and admission for hemorrhagic stroke (OR 1.97, 95% CI 1.71–2.26) were associated with more DNR orders. On the other hand, certain chronic comorbid diseases (cardiovascular and respiratory), sepsis (OR 0.89, 95% 0.82–0.98), admissions for postoperative purposes (OR 0.82, 95% CI 0.80–0.85), more recent admissions (OR for admissions in 2006–2011 period versus ≤2005 0.76, 95% CI 0.68–0.85 and OR for admissions in 2012–2017 period versus ≤2005 0.35, 95% CI 0.32–0.39) were associated with less DNR orders.

Among the 14531 patients who were intubated, 2660 patients (18.3%) had DNR orders. Among the 3706 patients who had renal replacement therapy, 958 patients (25.8%) had DNR orders.

### 3.3. Outcomes of Patients

Table 3 shows the outcomes of patients in the study cohort. ICU mortality was higher in patients with DNR orders (2167/3217 patients [67.8%] versus 1807/21573 patients [8.5%], *p* < 0.0001), with most deaths in the ICU (2186/3975 deaths, 55.0%) occurred with a DNR order. The overall hospital mortality was 26.5%, which was significantly higher in patients with DNR orders (2650/3217 patients [82.4%]) than in those with “full code” (3908/21573 patients [18.1%], *p* < 0.0001).  Figure 2 shows that the hospital mortality rate of patients who had DNR orders during ICU stay declined to its lowest in the 2011–2017 period.

Patients with DNR orders had more tracheostomies (15.2% versus 8.8%, *p* < 0.0001). The median duration of mechanical ventilation for DNR patients was five days (Q1, Q3: 2, 13) versus one day (Q1, Q3: 0, 5) for “full code” patients (*p* < 0.0001). The length of ICU stay was longer for the patients with DNR orders (median 6.3 days [Q1, Q3: 2.1, 13.7] versus three days [Q1, Q3: 1.1, 8.5], *p* < 0.0001). In contrast, the length of hospital stay was shorter in the patients with DNR orders (16 days [Q1, Q3: 7, 32] versus 23 days [Q1, Q3: 11, 50]).

## 4. Discussion

Our study showed that DNR orders were commonly practiced in a tertiary-care ICU in Saudi Arabia but were less frequent after 2005 compared to the 1999–2005 period. The main determinants of a DNR order were older age, poor pre-morbid functional status, higher critical illness severity, and hemorrhagic stroke. Patients with sepsis or surgical admission were less likely to have DNR orders. DNR orders preceded most deaths (55.0%) in the ICU. Patients with DNR orders had higher mortality than patients with full code, but the mortality declined in more recent years.

Practices of withholding life support in Saudi Arabia are based on an Islamic fatwa from 1988 [16] and the code of ethics for healthcare practice of the Saudi Commission for Health Specialties [15]. In the current study, DNR orders were implemented in 13% of patients in the ICU. This rate is similar to what had been observed in other studies from different countries, including Saudi Arabia [17, 18]. In an international study, a decision to withhold/withdraw life support during the ICU stay was reported in 1259/9524 (13%) patients [9]. A multicenter study in 40 ICUs in the United States found that 9% of admissions (1988–1990) had DNR orders written in the ICU (range, 1.5 to 22%) [17]. A study from an ICU in Saudi Arabia found that 14.9% of all ICU discharges (January 1 to December 31, 2021) had DNR orders [18]. However, the practice of DNR is more common in other ICUs. A systematic review found an overall range of DNR orders from 5.4% to 82.0% based on data from 36 studies [19]. One factor that may partially explain the relatively low prevalence of DNR orders in our study is that advance directives were not practiced in our hospital during the study period.

Patient and non-patient factors may affect decisions regarding the goals of care and life support. We found that older age, poor baseline functional status and high illness severity were associated with DNR orders. Similar findings were reported by other studies [19–22]. We also found that surgical patients were less likely to have DNR orders. This also has been observed by other investigators [19]. In our study, patients with certain chronic comorbidities were less likely to have DNR orders. This is against the findings of other studies [17, 19] but has been observed in others [23]. This finding should be interpreted cautiously and may be due to a selection bias because only patients who requested fullsupport were admitted to our ICU. In recent years, we found a lower prevalence of DNR orders. This opposed published evidence where there was a trend for increasing use of limitation of life support measures over time [4, 19]. However, there is variation in these practices between ICUs [2–5, 7–9, 19, 21]. Differences in preferences and practices among ICU physicians and differences in culture from one ICU to another may influence end-of-life decision-making [21, 24].

ICU and hospital mortality rates were higher for patients with DNR orders when compared to those with a “full code”. This finding was expected. We found that 55% of the deceased patients in the ICU had DNR orders before death. This rate is lower than that reported in other studies [18, 25]. In a study of a large US sample of ICU patients (400129 admissions, 9891 deaths in the ICU), 91.3% of patients had DNR order before death [25]. In the current study, there was an improvement in the survival at hospital discharge of patients with DNR orders. This may be related to organizational changes, staffing, patient mix and preferences, and clinical care in the hospital and ICU during the study period. For example, a critical care response team was implemented in the hospital in 2006 [14] and a sepsis response team in the emergency department in 2013 [26]. These changes were associated with lower APACHE II scores for patients at ICU admission and lower mortality [14, 26].

The study findings should be interpreted in light of its strengths and limitations. Our study included a large number of patients who had different characteristics and were admitted over 19 years. These allow for a good characterization of the practices of DNR orders. The study limitations include its retrospective design and being performed in one center, and thus the results cannot be generalized to other ICUs in Saudi Arabia or the region. Relevant data regarding the patients who deemed DNR appropriate but remained “full code” due to the surrogate decision makers' refusal were unavailable. Lastly, this study did not explore social and other factors that may be associated with end-of-life care, such as race, and education and income levels. We also did not evaluate the opinions of different physicians regarding withholding life support and its impact on DNR orders.

## 5. Conclusions

DNR orders were commonly practiced in patients admitted to a tertiary-care ICU in Saudi Arabia between 1999 and 2017. Baseline patient factors that affected this practice included older age, poor baseline functional status, and a higher severity of illness. Patients with DNR orders had higher mortality than those with “full code,” but their hospital survival increased in recent years, reaching approximately 15%. The present study can help clinicians in decision making regarding life support in critically ill patients.

## Figures and Tables

**Table 1:** Baseline characteristics of patients with do-not-resuscitate (DNR) status and patients with full code status.

Variables	All *N* = 24790	Do-not-resuscitate (DNR) *N* = 3217	Full code *N* = 21573	*p* value
Demographics				
Age (yrs), median (Q1, Q3)	58 (35, 72)	67 (55, 76)	57 (33, 71)	<0.0001
Male sex, *n* (%)	15216 (61.4)	1843 (57.3)	13373 (62)	<0.0001
Female sex, *n* (%)	9574 (38.6)	1374 (42.7)	8200 (38.0)	
Pre-morbid WHO performance status score^*∗*^				
0	11847 (47.8)	934 (29.1)	10913 (50.6)	<0.0001
1	3744 (15.1)	467 (14.5)	3277 (15.2)	
2	3681 (14.9)	624 (19.4)	3057 (14.2)	
3	3013 (12.2)	584 (18.2)	2429 (11.3)	
4 and 5	2500 (10.1)	606 (18.9)	1894 (8.8)	
Admission category, *n* (%)				
Respiratory	4754 (19.2)	598 (18.6)	4156 (19.3)	<0.0001
Cardiovascular^*∗∗*^	8507 (34.4)	1729 (53.8)	6778 (31.5)	
Neurologic	2168 (8.8)	318 (9.9)	1850 (8.6)	
Other medical	1391 (5.6)	206 (6.4)	1185 (5.5)	
Nonoperative trauma	2673 (10.8)	120 (3.7)	2553 (11.9)	
Postoperative	5273 (21.3)	246 (7.7)	5027 (23.3)	
Sepsis, *n* (%)	9488 (38.3)	1539 (47.8)	7949 (36.9)	<0.0001
Ischemic stroke, *n* (%)	2603 (10.5)	530 (16.5)	2073 (9.6)	<0.0001
Hemorrhage stroke, *n* (%)	2789 (11.3)	325 (10.1)	2464 (11.4)	0.03
Chronic comorbidities, *n* (%)^*∗∗∗*^				
Chronic cardiovascular disease	4807 (19.5)	753 (23.6)	4054 (18.9)	<0.0001
Chronic liver disease	1938 (7.9)	616 (19.3)	1322 (6.2)	<0.0001
Chronic respiratory disease	3457 (14.01)	469 (14.7)	2988 (13.9)	0.23
Chronic renal disease	2531 (10.3)	516 (16.2)	2015 (9.4)	<0.0001
Immunocompromised status^*∗∗∗∗*^	2516 (10.2)	595 (18.6)	1921 (8.9)	<0.0001
Diabetes	8952 (36.1)	1379 (42.9)	7573 (35.1)	<0.0001
Laboratory and clinical findings in the first 24 hours				
APACHE II score, median (Q1, Q3)	20 (14, 27)	28 (23, 34)	19 (13, 14)	<0.0001
Bilirubin (mmol/L), median (Q1, Q3)	14 (9, 29)	21 (11, 58)	14 (8, 27)	<0.0001
GCS, median (Q1, Q3)	13 (8, 15)	8 (3, 13)	14 (9, 15)	<0.0001
Creatinine (*μ*mol/L), median (Q1, Q3)	89 (61, 174)	139 (80, 248)	84 (60, 159)	<0.0001
Lactic acid, median (Q1, Q3)	2 (1.2, 3.6)	3.1 (1.7, 6.9)	1.9 (1.2, 3.3)	<0.0001
INR, median (Q1, Q3)	1.2 (1.1, 1.5)	1.4 (1.2, 2.1)	1.2 (1.1, 1.5)	<0.0001
PaO_2_/FiO_2_ ratio, median (Q1, Q3)	235 (148, 342)	173 (107, 267)	243 (155, 348)	<0.0001
Therapies during ICU stay				
Mechanical ventilation, *n* (%)	14531 (58.6)	2660 (82.7)	11871 (55.0)	<0.0001
Renal replacement therapy, *n* (%)	3706 (14.95)	958 (29.8)	2748 (12.7)	<0.0001
Admission year				
≤2005	4915 (19.8)	932 (29)	3983 (18.5)	<0.0001
2006–2011	6079 (24.5)	994 (30.9)	5085 (23.6)	
2012–2017	13796 (55.7)	1291 (40.1)	12505 (58)	

APACHE II: acute physiology and chronic health evaluation II; GCS: glasgow coma score; INR: internal normalized ratio; PaO_2_/FiO_2_ ratio: the ratio of the partial pressure of oxygen to the fraction of inspired oxygen. ^*∗*^0: able to carry out all normal activity without restriction; 1: restricted in strenuous activity but ambulatory and able to carry out light work; 2: ambulatory and capable of all self-care but unable to carry out any work activities; up and about more than 50% of waking hours; 3: symptomatic and in a chair or in bed for greater than 50% of the day but not bedridden; 4: completely disabled; cannot carry out any self-care; totally confined to bed or chair; 5: dead; Q1: first quartile, Q3: third quartile; For all percentages, the denominator is the total number of subjects in the group. Continuous variables were compared using *T*-Test and categorical value using Chi-square test. ^*∗∗*^Cardiovascular reasons for ICU admission as defined by the APACHE II system include cardiovascular failure of insufficiency due to hypertension, rhythm disturbance, hemorrhagic shock/hypovolemia, coronary artery disease, sepsis, postcardiac arrest, cardiogenic shock and pulmonary embolism. ^*∗∗∗*^One patient may have more than one comorbid condition. ^*∗∗∗∗*^defined as receiving therapy that suppresses resistance to infection (e.g., immunosuppression, chemotherapy, radiation, long-term or high-dose steroids, or advanced leukemia, lymphoma, cancer, or AIDS).

**Table 2:** Predictors of do-not-resuscitate (DNR) status (multivariable logistic regression analysis). The independent variables entered in the model were age, premorbid WHO performance status, admission category, sepsis, ischemic stroke, hemorrhagic stroke, chronic cardiovascular disease, chronic renal disease and chronic respiratory disease, APACHE II and admission year.

Variables	OR	95% confidence	*p* Value
AGE (for every 1-year increase)	1.01	1.01-1.02	<0.0001
Pre-morbid WHO performance status			
1 vs 0	1.27	1.11–1.45	0.0005
2 vs 0	1.48	1.30–1.68	<0.0001
3 vs 0	1.67	1.46–1.92	<0.0001
4 vs 0	2.48	2.16–2.86	<0.0001
Postoperative admission vs. nonoperative admission	0.82	0.80–0.85	<0.0001
Sepsis	0.89	0.82–0.98	0.01
Ischemic stroke	1.12	0.99–1.26	0.06
Hemorrhagic stroke	1.97	1.71–2.26	<0.0001
Chronic cardiovascular disease versus others	0.83	0.75–0.92	0.0003
Chronic respiratory disease versus others	0.64	0.57–0.72	<0.0001
Chronic renal disease versus others	0.88	0.78–0.99	0.03
APACHE II (for every 10-unit increase)	1.09	1.08–1.10	<0.0001
Admission year			
2006–2011 vs ≤2005	0.76	0.68–0.85	<0.0001
2012–2017 vs ≤2005	0.35	0.32–0.39	<0.0001

OR: odd ratio. APACHE II: acute physiology and chronic health evaluation II.

**Table 3:** Outcome of patients with do-not-resuscitate (DNR) status and those with full code status.

Variables	All *N* = 24790	Do-not-resuscitate (DNR) *N* = 3217	Full code *N* = 21573	*p* value
ICU mortality, *n* (%)	3975 (16.2)	2168 (67.8)	1807 (8.5)	<0.0001
Hospital mortality, *n* (%)	6558 (26.5)	2650 (82.4)	3908 (18.1)	<0.0001
Tracheostomy, *n* (%)	2378 (9.6)	489 (15.2)	1889 (8.8)	<0.0001
Mechanical ventilation duration (days), median (Q1, Q3)	2 (0, 6)	5 (2, 13)	1 (0, 5)	<0.0001
ICU LOS (days), median (Q1, Q3)	3.4 (1.2, 9.2)	6.3 (2.1, 13.7)	3.1 (1.1, 8.5)	<0.0001
Hospital LOS (days), median (Q1, Q3)	21 (10, 47)	16 (7, 32)	23 (11, 50)	<0.0001

ICU: intensive care unit; LOS: length of stay.

## Data Availability

The data from this study will be made available upon request to the corresponding author and in accordance with the regulations of King Abdullah International Medical Research Center.
